# Supplementary material for: Transcriptome diversity is a systematic source of variation in RNA-sequencing data
Source: PLoS Comput Biol. 2022 Mar 24;18(3):e1009939. doi: 10.1371/journal.pcbi.1009939 (PMC8982896; doi:10.1371/journal.pcbi.1009939)
Supplement: S1 File — Supplementary figures (Figs A–J). Fig A in S1 File. Transcriptome diversity is highly correlated with within-sample gene expression variance in both TMM and TPM estimates. Transcriptome diversity across samples from a large RNA-seq study in D. melanogaster [16] shows significant associations with gene expression variance both in TMM estimates (left) and TPM (right). Variance was computed using TMM and TPM values respectively. Spearman correlation coefficients and p-values were computed and shown in each panel. Fig B in S1 File. Transcriptome diversity is associated with the PCs across human tissues related to Fig 4A. For each GTEx tissue, the dot plot shows the absolute Spearman correlation coefficient between transcriptome diversity values and the loadings of a PC from a PCA performed on the full TMM expression matrix. To the right, the directionality of the correlation is shown (+/-) along with the PC used and its total variance explained. The PC with the highest correlation with transcriptome diversity is shown. Tissues are ordered by sample size. Fig C in S1 File. Gene length, GC content and gene expression level are associated with the correlation of gene expression with transcriptome diversity. For each GTEx tissue, -10*log10(p-value) was computed from a multiple regression of association level of gene expression to transcriptome diversity on gene length, GC content and gene expression level. For visualization purpose, 1e-50 was added to all p-values. The black dashed line shows the cut-off p-value equal to 0.05. a In TMM estimates, lower average gene expression tended to have stronger association between gene expression and transcriptome diversity across all tissues. GC content showed negative correlation in most tissues, i.e. lower GC content has stronger association, except positive correlation observed in 7 tissues (adipose subcutaneous, artery aorta, brain cortex, breast mammary tissue, pancreas, thyroid and uterus). Gene length showed significant associa [file pcbi.1009939.s001.pdf]

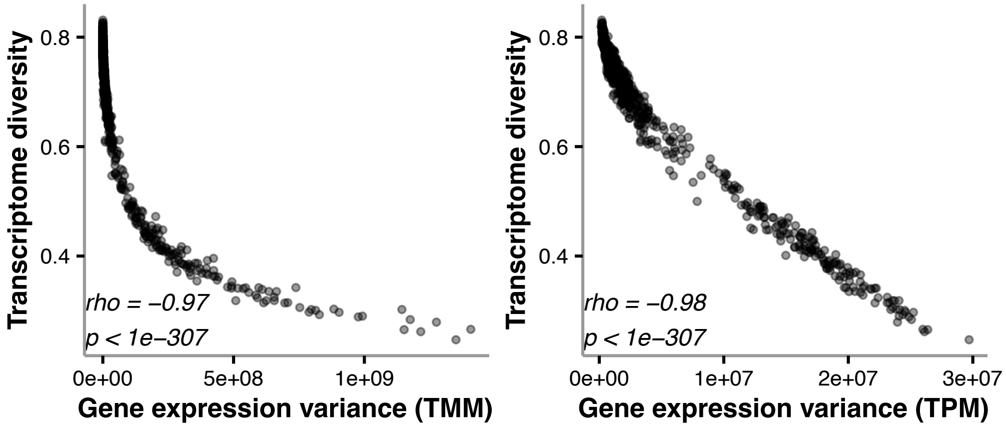

Fig A. Transcriptome diversity is highly correlated with within-sample gene expression variance in both TMM and TPM estimates. Transcriptome diversity across samples from a large RNA-seq study in *D. melanogaster* [16] shows significant associations with gene expression variance both in TMM estimates (left) and TPM (right). Variance was computed using TMM and TPM values respectively. Spearman correlation coefficients and p-values were computed and shown in each panel.

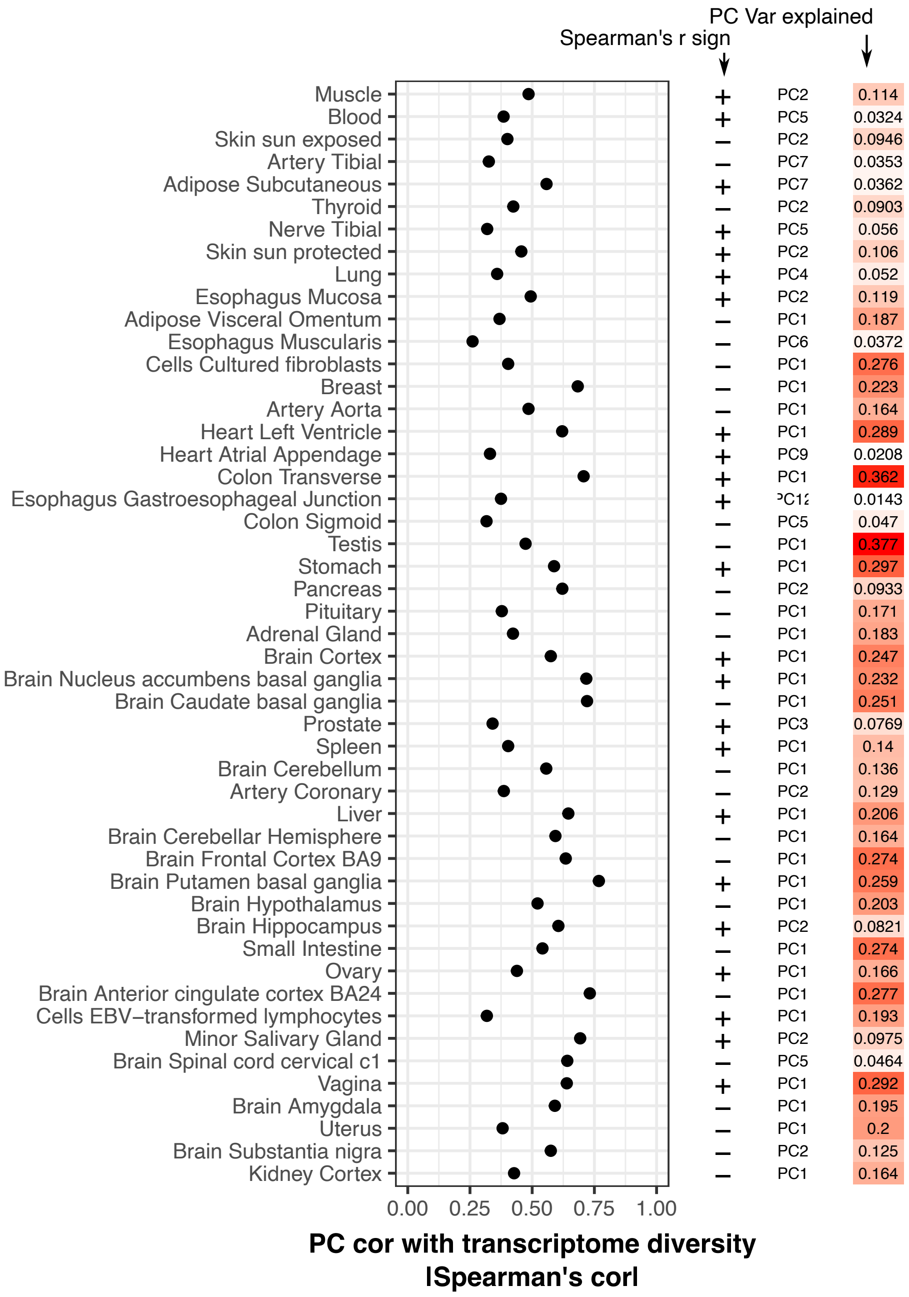

Fig B. Transcriptome diversity is associated with the PCs across human tissues related to Fig 4A. For each GTEx tissue, the dot plot shows the absolute Spearman correlation coefficient between transcriptome diversity values and the loadings of a PC from a PCA performed on the full TMM expression matrix. To the right, the directionality of the correlation is shown (+/-) along with the PC used and its total variance explained. The PC with the highest correlation with transcriptome diversity is shown. Tissues are ordered by sample size.

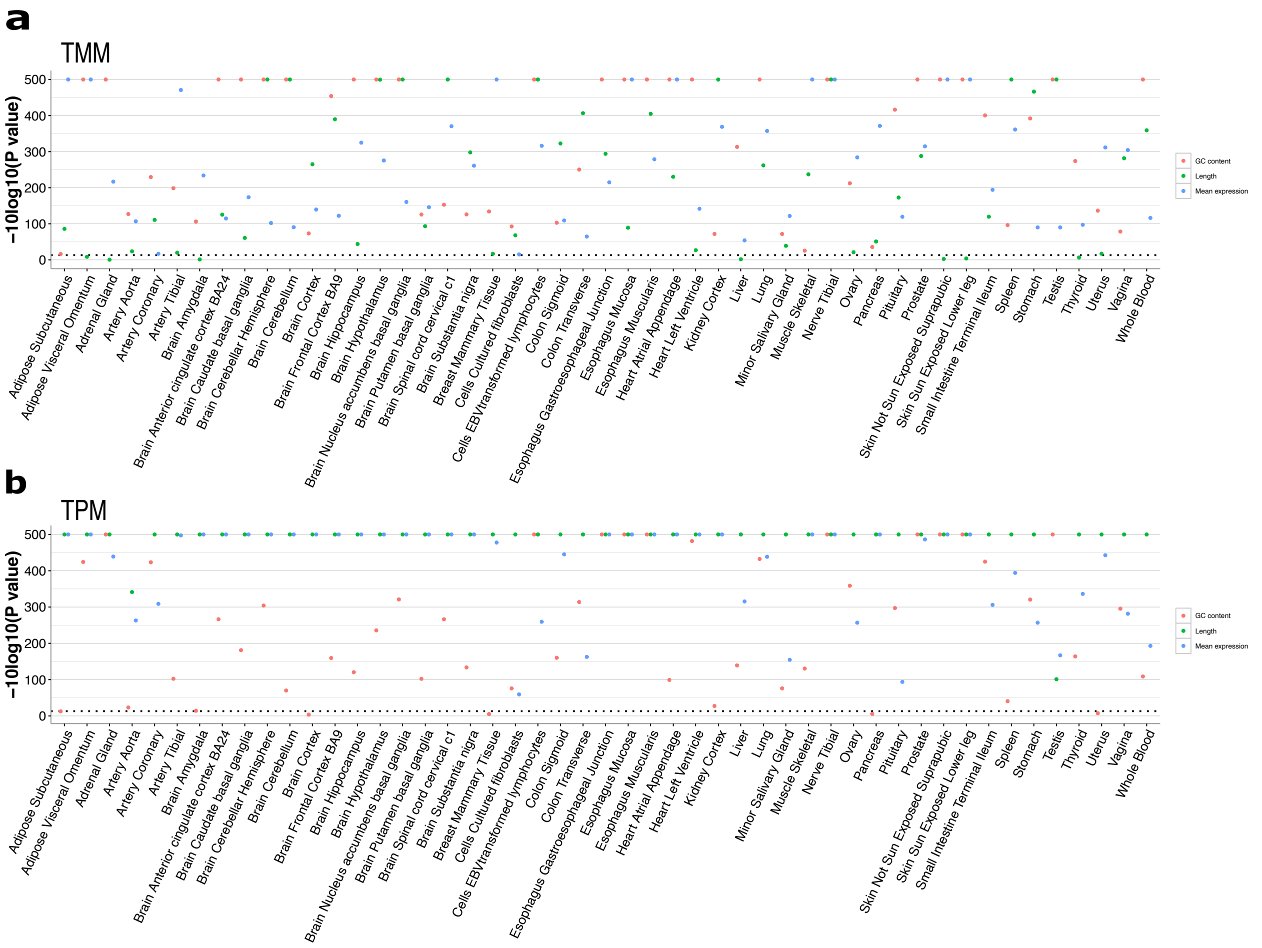

Fig C. Gene length, GC content and gene expression level are associated with the correlation of gene expression with transcriptome diversity. For each GTEx tissue, -10*log10(p-value) was computed from a multiple regression of association level of gene expression to transcriptome diversity on gene length, GC content and gene expression level. For visualization purpose, 1e-50 was added to all p-values. The black dashed line shows the cut-off p-value equal to 0.05. **a** In TMM estimates, lower average gene expression tended to have stronger association between gene expression and transcriptome diversity across all tissues. GC content showed negative correlation in most tissues, i.e. lower GC content has stronger association, except positive correlation observed in 7 tissues (adipose subcutaneous, artery aorta, brain cortex, breast mammary tissue, pancreas, thyroid and uterus). Gene length showed significant association in 42 out of 49 tissues (except adipose visceral omentum, adrenal gland, brain amygdala, liver, skin not sun exposed suprapubic, skin sun exposed lower leg and thyroid), and longer genes showed stronger association in most tissues (except artery tibial, brain amygdala, skin not sun exposed suprapubic and testis). **b** In TPM estimates, longer genes and lower average gene expression tended to have stronger association between gene expression and transcriptome diversity across all tissues except that gene length showed negative correlation in testis. GC content showed significant association in 44 out of 49 tissues (except adipose subcutaneous, brain cortex, breast mammary tissue, pancreas and uterus), and most correlations between GC content and diversity association are negative except artery aorta, brain cortex, breast mammary tissue and thyroid.

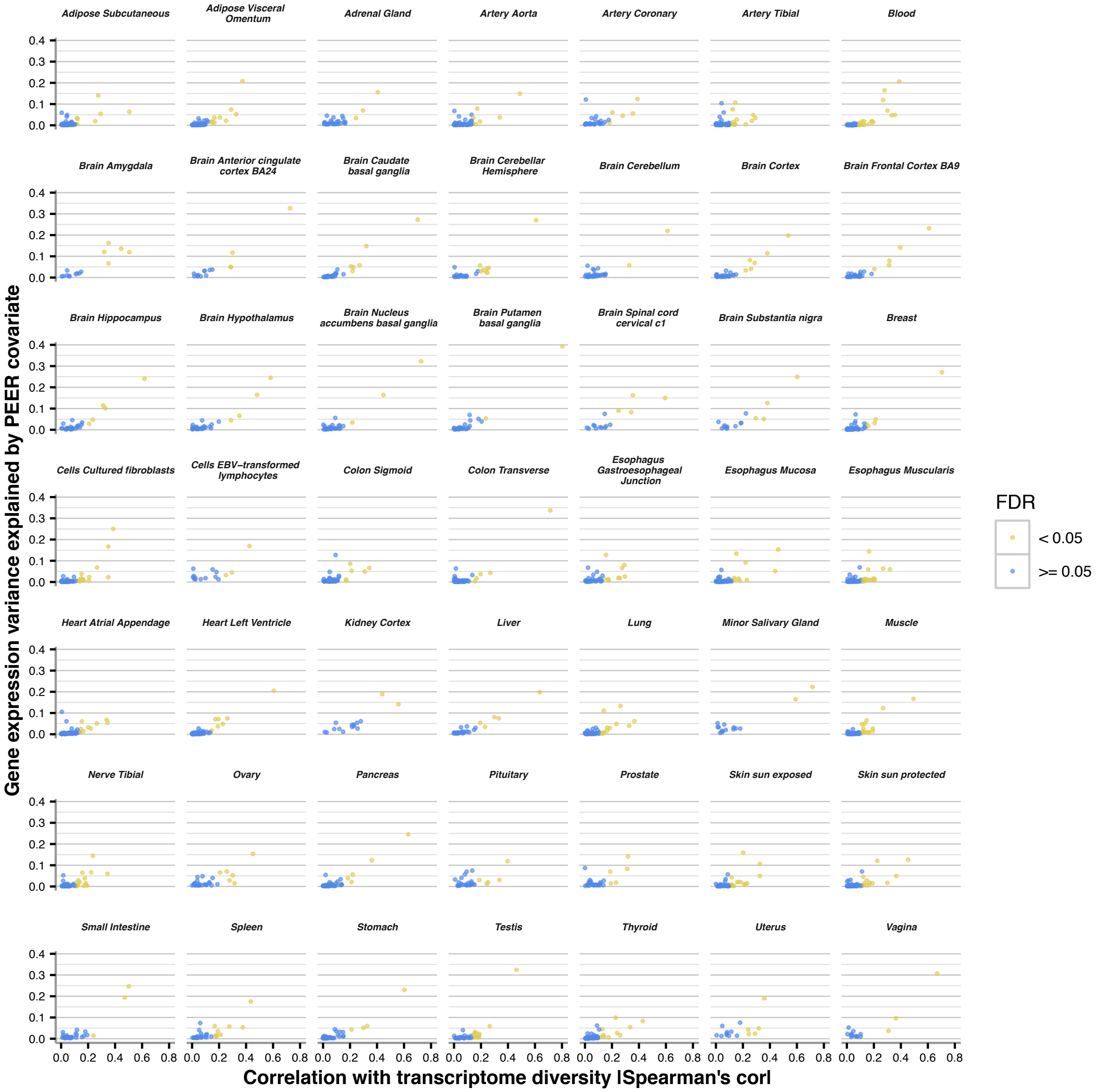

Fig D. PEER covariates associated with transcriptome diversity explain a large fraction of variance in global gene expression. For each tissue, the Spearman correlation coefficient between transcriptome diversity values and the values of all PEER covariates were computed and colored by significance of correlation using BH-FDR (BH-FDR >= 0.05 in blue, BH-FDR < 0.05 in yellow). The variance of the full expression matrix explained by each PEER covariate was computed and projected on the y axis.

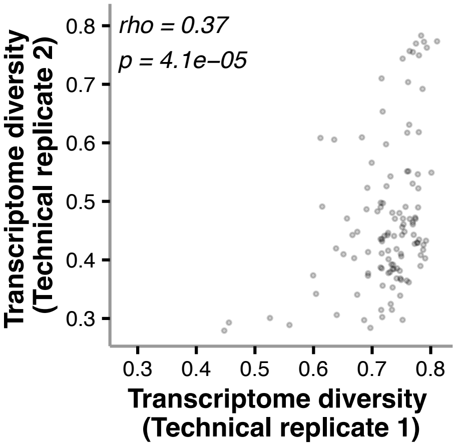

Fig E. Variation observed in transcriptome diversity among technical replicates. 117 flies with 2 technical replicates were tested by Lin et al. (2016) [16]. For each pair of technical replicates, samples with higher transcriptome diversity values were assigned into technical replicate 1 and samples with lower transcriptome diversity were assigned into technical replicate 2. Spearman correlation coefficient and p-value were computed and shown.

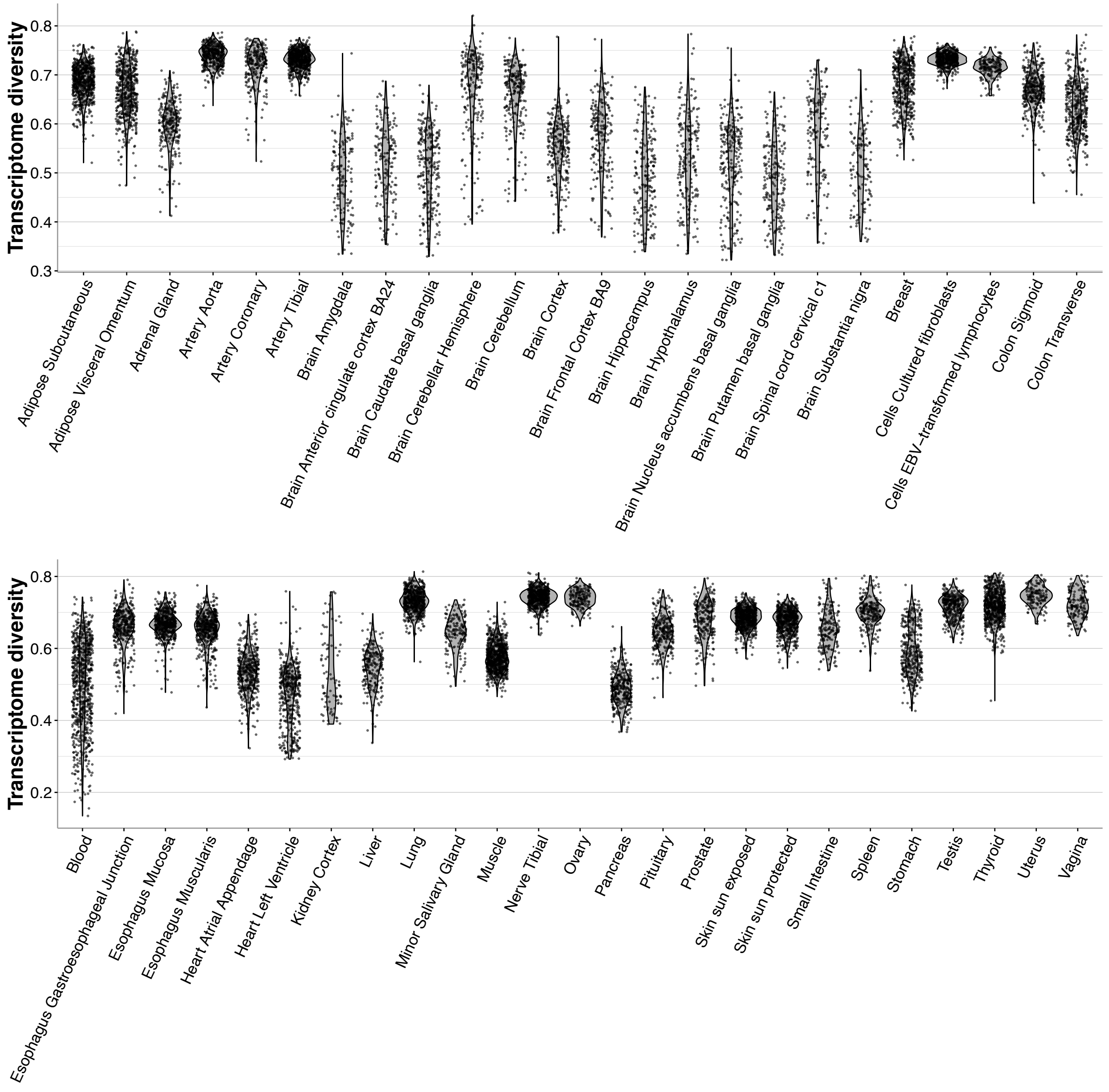

Fig F. Large variation observed in transcriptome diversity across tissues in GTEx. Transcriptome diversity values' distribution are shown in violin plots for all tissues in GTEx, indicating a wide range of variation for transcriptome diversity among tissues.

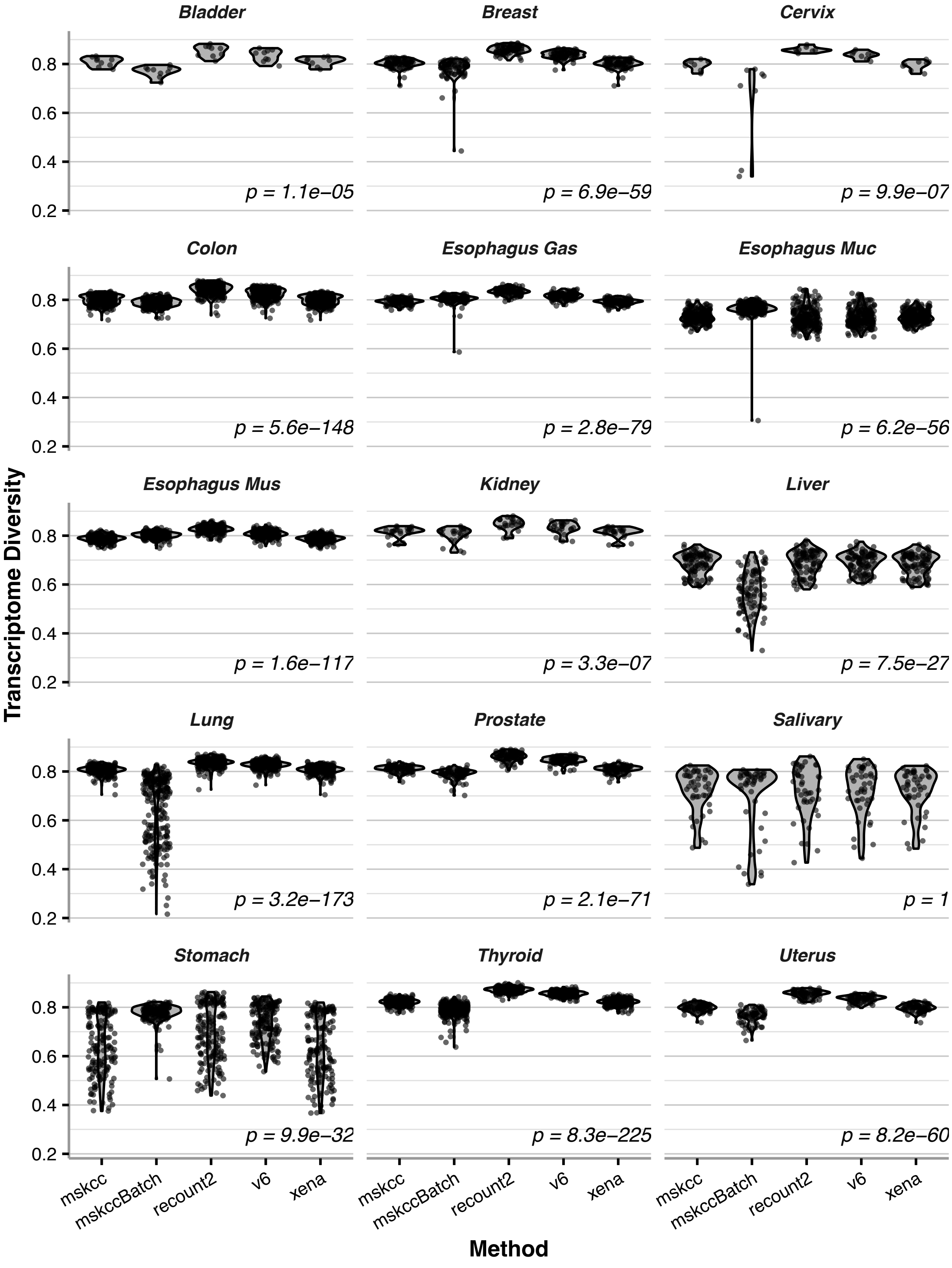

Fig G. Differences on RNA-seq computational pipelines have a strong impact on transcriptome diversity. Five computation pipelines for RNA-seq data (mskcc, mskccBatch, recount2, v6 and xena) are shown to have impacts on transcriptome diversity across tissues (data from Arora et al. (2020) [19]). Kruskal-Wallis rank sum tests were performed, and p-values are shown in each panel. 14 out of 15 tissues (all except salivary) showed significant differences in the distributions of transcriptome diversity values among the five pipelines.

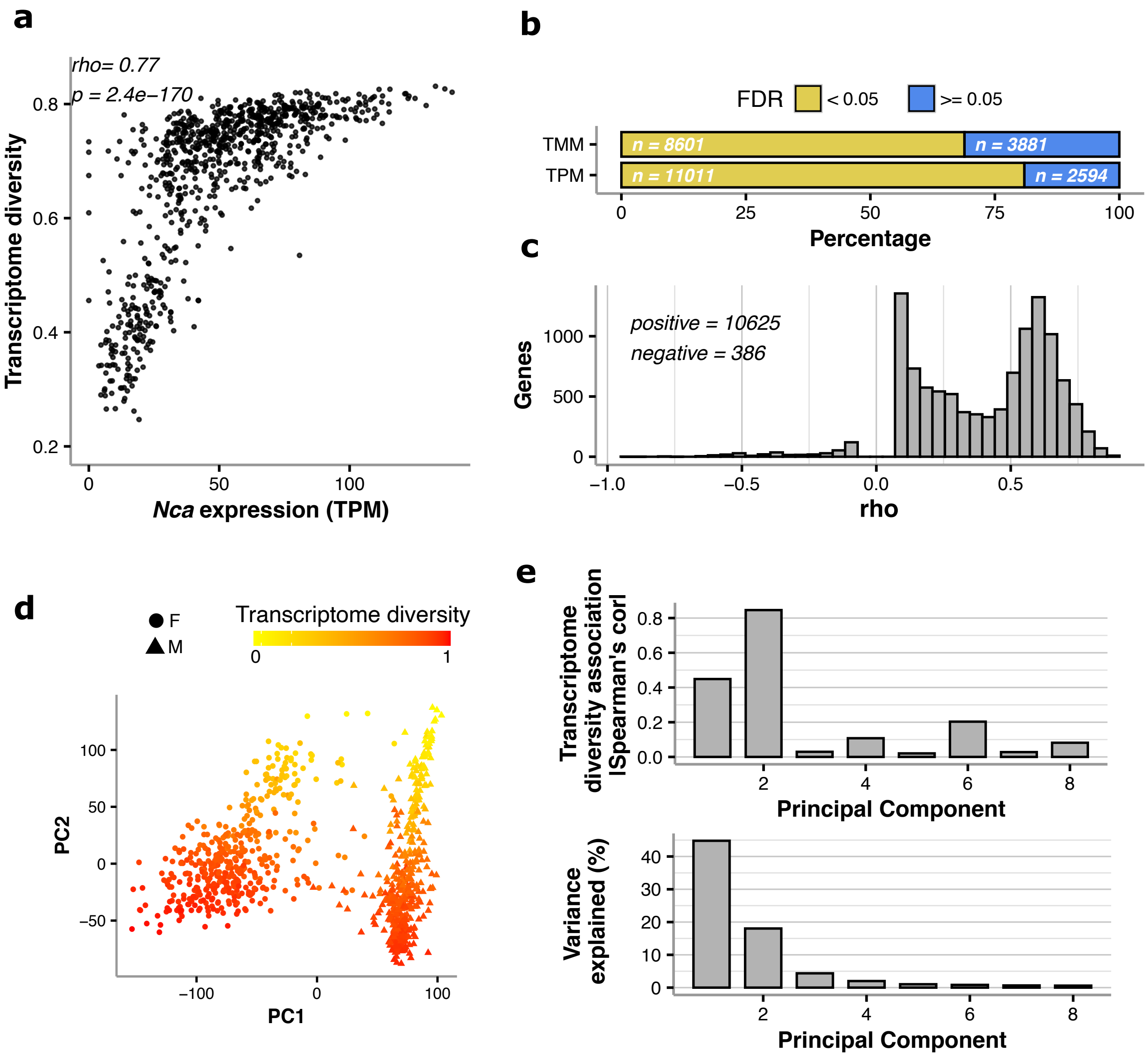

Fig H. Transcriptome diversity is associated with global gene expression in *D. melanogaster*, similar analysis on TPM estimates related to Fig 2. a Example of a strong association between the TPM expression of a gene (*Nca*) and transcriptome diversity across samples from a large RNA-seq study [16]. **b** Percentage of genes whose expression was significantly associated with transcriptome diversity (as in *a*; BH-FDR < 0.05 in yellow) vs those that were not (BH-FDR >= 0.05 in blue). The actual number of genes is shown with white text. **c** Most significant associations using TPM estimates are positive, as shown here by the distribution of Spearman's correlation coefficients (rho) between transcriptome diversity and gene expression. **d** Loadings from the first two principal components (PCs) from a principal component analysis done on the full TPM expression matrix; samples are colored by transcriptome diversity and the point shape corresponds to sex. **e** Absolute Spearman's correlation coefficients between transcriptome diversity and loadings of the first 8 PCs (top), and variance explained by each of those PCs of the full expression matrix.

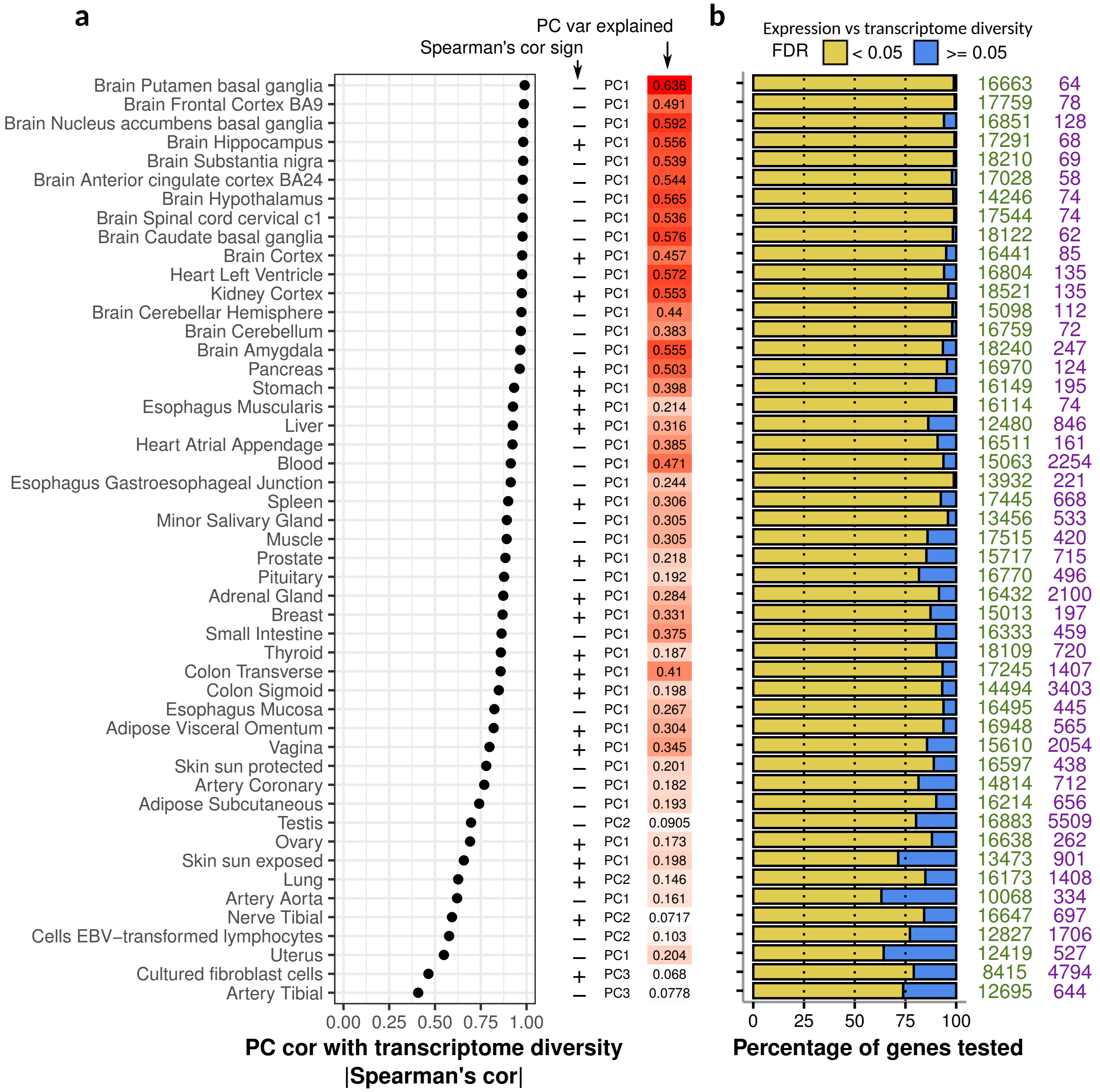

Fig I. Transcriptome diversity is associated with the expression of most genes across human tissues, similar analysis on TPM estimates related to Fig 4. a For each GTEx tissue, the dot plot shows the absolute Spearman correlation coefficient between transcriptome diversity values and the loadings of a PC from a PCA performed on the full TPM expression matrix. To the right, the directionality of the correlation is shown (+/-) along with the PC used and its total variance explained. The PC with the highest correlation with transcriptome diversity is shown. **b** For each tissue, the percentage of genes whose expression TPM was significantly associated with transcriptome diversity (as in *a*; BH-FDR < 0.05 in yellow) vs those that were not (BH-FDR >= 0.05 in blue), the numbers on the right represent the directionality of the significant correlations (green are positive significant associations, and purple are negative significant associations). Significance was assessed using a linear regression approach (see Methods).

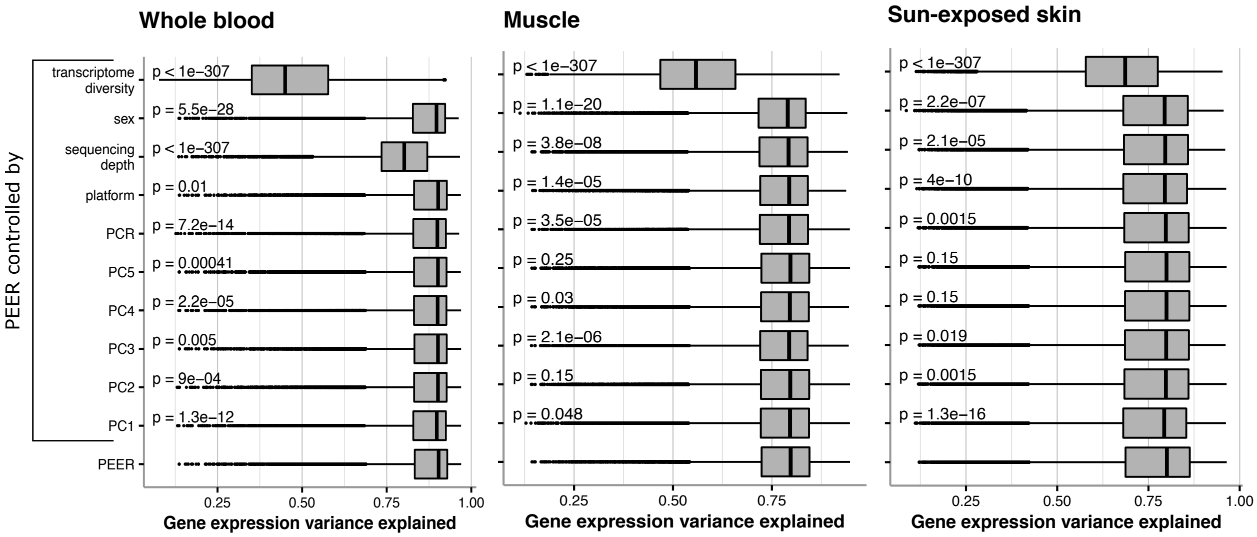

Fig J. In GTEx PEER covariates correlate with transcriptome diversity on TPM estimates related to Fig 5B. Identical to Fig 5B but for whole blood, muscle and sun-exposed skin GTEx samples in TPM estimates. Boxplots showing the distribution of variance explained values (*r^2*) from linear regressions done on the expression of each gene using either intact PEER covariates, or the residuals of regressions performed on the same PEER covariates using the variables shown (controlled PEER rows). Mann-Whitney tests against the intact PEER covariates were performed for each of the controlled PEER distributions and the corresponding p-values are shown.
