# Supplementary material for: Transcriptome diversity is a systematic source of variation in RNA-sequencing data
Source: PLoS Comput Biol. 2022 Mar 24;18(3):e1009939. doi: 10.1371/journal.pcbi.1009939 (PMC8982896; doi:10.1371/journal.pcbi.1009939)
Supplement: Attachment — Submitted filename: reply_to_reviewer.pdf [file pcbi.1009939.s008.pdf]

Reviewer #1: This paper introduces the concept of transcriptome diversity and defines it as the Shannon entropy of gene expression values within a given sample. The authors argue for importance of the concept from a few different aspects. The main take-away message is that PEER factors and PCs can be largely explained by transcriptome diversity, which authors believed to be largely correlated with confounders. Although as I understand it this message would not change current practices, this message may be informative to computational researchers.

I find the paper well-written overall. I provide some detailed suggestions below. I hope the authors can organize their work better and make the paper more valuable to the field.

Major points:
1. Specific subsections within MATERIALS AND METHODS should be referenced in main text and figure captions when relevant. For example, the paragraph starting in Line 201 uses the method described in Line 579 (I think), but it wasn't specifically referenced. There are many instances like this throughout the results section.

We have added the suggested reference and corrected this in other places too.

2. In the current manuscript, sometimes the analyses are directly based on TPM or TMM-based expression values, other times the values are rankit-normalized before the analyses. Can these two approaches be unified to simplify the methodology? At the very least, it should be made much clearer in RESULTS and the figure captions which approach is used in each case, and the reasoning should be provided.

Thanks for the suggestions, we have modified the manuscript to 1) Focus only on TMM-based values, and 2) State the reasoning when using rankit normalization. We have also modified the Figure captions to make this clear.

3. As shown in Fig. 2c, 96% of the TPM significant correlations are positive. Could the authors provide some explanations for this phenomenon?

We have modified the manuscript to focus on analysis of TMM estimates and the ratio was decreased to 64% positive correlation. One possible explanation is that due to a small number of very highly expressed genes, most genes are expressed below the mean level in any given sample. Therefore increasing the expression of most genes will tend to increase the diversity.

4. For Figure 4, ordering the tissue types by sample size would be informative. Currently the tissue types are ordered by absolute value of Spearman correlation, it seems like. Ordering them by sample size may reveal additional pattern.

We did try reordering this figure by sample size (shown below), but we didn't see any clear pattern.

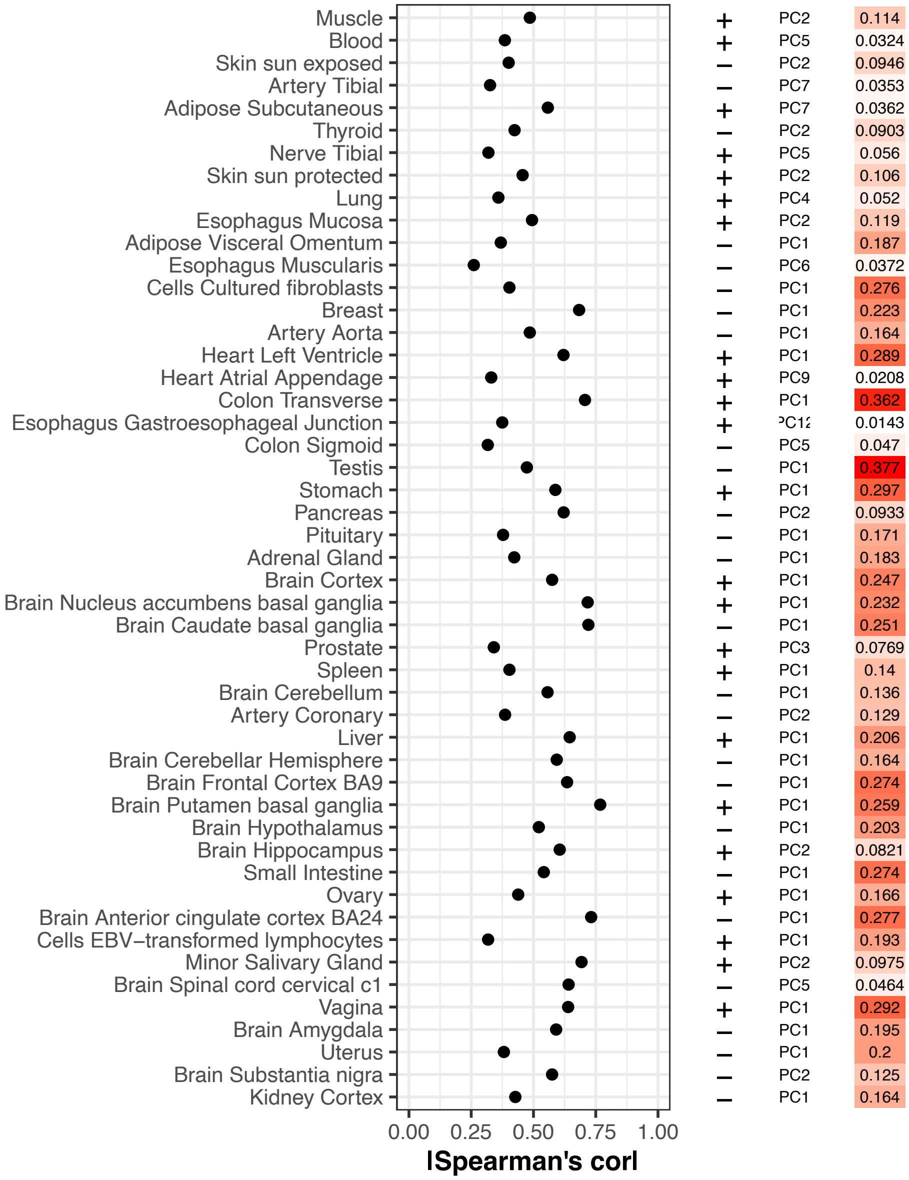

5. Is there any difference in transcriptome diversity for technical replicates?

Yes, in Lin et al dataset, as shown below, variation in transcriptome diversity values were observed among technical replicates. We have added description of this observation to main text and this figure to supplementary figures to demonstrate that there are differences in transcriptome diversity for technical replicates.

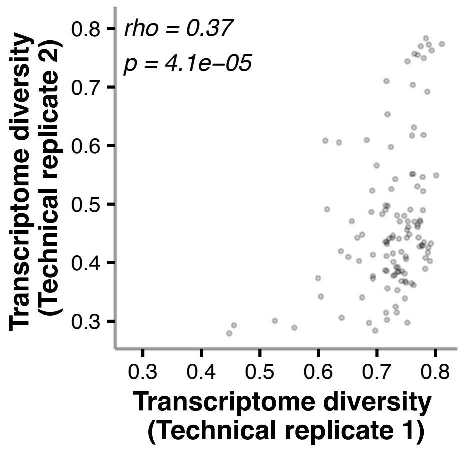

6. For this manuscript, the authors treat the transcriptome diversity as a systematic source of bias in RNA-seq data. However, the difference of transcriptome diversity across samples itself may have some biological meaning. The authors should discuss this possibility.

Thanks for the suggestion. We agree that transcriptome diversity may not be pure bias. We have changed the title to remove the word “bias” and added a new paragraph about this to the Discussion section.

7. As stated by the authors, TPM values have been shown to not properly account for sequencing depth differences across samples and for the influence of highly expressed genes on the rest of genes and are not proper for cross-sample comparison. Thus, the results from TMM-based expression values should be given more emphasis in the manuscript.

We have moved the TMM-based analysis to the main text in order to emphasize those, and moved TPM-based results to the supplementary material.

Minor points:

1. The authors should clarify how they calculate the gene expression variance in Fig. S1 and make use of “variance” or “variation” consistently between the x-axis label and the figure title.

Variance was computed using TPM and TMM values respectively. The description has been added to the legend.

2. For Fig. 6a-c, the gray-black color scale is not distinguishable (the scale is too subtle).

Figures have been updated.

3. The points and violin plots in some figures of Fig. S7 and S8 overlap. Please adjust the width or transparency of the points.

The width of violin points of Fig. S7 and S8 has been adjusted.

4. Figure 2b and 2c: expression and transcriptome diversity are rankit-normalized? And r in the caption stands for Pearson correlation? Details like this should be made self-evident in the captions.

We have modified this figure and its caption to make it self-evident.

5. For MATERIALS AND METHODS section:
a. Line 469, Transcriptome diversity calculation (Shannon entropy)
i. It might be helpful to explain briefly why H ranges from 0 to log2(G) for readers who are not experts in information theory.

Details about why H ranges from 0 to log2(G) were added to the Methods section: "***H*** ranges from *0 to $log_2(G)$,* where ***H*** = 0 when all transcripts are from only one gene and ***H*** = *$log_2(G)$* when an equal number of transcripts are measured across all genes.".

ii. I think it would be more straightforward to define p_i directly in terms of TPM, i.e., using the third equation on the first page of Additional File 4: Note S1, since a TPM value represents the relative abundance of a gene in a sample, the read lengths of genes already accounted for. More generally, p_i should be defined in terms of the normalized gene expression values, which would encompass TMM normalized values as well.

We now define p_i in terms of TPM in the main text. We do not think it would be ideal to define p_i using TMM since this across-sample normalization would make the transcriptome diversity values depend on what other samples are being included and therefore more difficult to interpret.

b. Line 549, PCA and clustering analysis:
i. The authors did the PCA analysis without scaling and centering. Are there any specific considerations for not using scaling and centering? In general, centering is almost always mandatory, and scaling is almost always preferred.

Thanks for pointing this out. We were doing PCA with centering and scaling, so this was mistakenly written and has been corrected.

c. Line 570, Gene expression variance explained by PEER accounted by transcriptome diversity
i. In Line 575, the sentence "The difference of r2 values from expression associations between the intact PEER vs the "regressed out" PEER factors divided by the variance explained by PEER represents the amount of variance explained by PEER that can be accounted for by transcriptome diversity" needs to be rewritten. The word "between" does not make sense. And does "variance explained by PEER" refer to the R2 value between a gene expression vector and the intact PEER factors? This section may be better if the authors used mathematical notations and equations.

Mathematical notations and more details have been added to clarify this section. And the order of the two sections ('Variance explained of gene expression matrices' and 'Gene expression variance explained by PEER accounted by transcriptome diversity') was switched to better aid understanding.

ii. This section seems to underly method behind Figure 5b. This section should be expanded to explain the methodology behind Figure 5b more comprehensively.

The title of this section was expanded to include other covariates, and more details about the methodology behind Figure 5b were added.

d. Line 579, Variance explained of gene expression matrices:
i. The description in this subsection would be made clearer if mathematical notations and equations are used.

Mathematical notations and more details were added.

Reviewer #2: The authors proposed a simple metric based on Shannon entropy to study the diversity of expression across genes within each sample. Through analyses, they found that the diversity measure is correlated with several biological variables and claimed it is a systematic source of bias. Below are some comments.

While it appears to be empirically correlated, I found it is hard to interpret the so-called bias. How does transcriptome diversity necessarily cause bias?

Fig 4a, the brain tissues are among the top ones with the highest absolute Spearman's correlation between PC (principal component) and transcriptome diversity. Is there any biological reason for this? The clustering of brain tissues may be a sign that "transcriptome diversity" may not be pure bias, and there may be some underlying biological meaning.

Thanks for the suggestion. We agree that transcriptome diversity may not be pure bias. We have changed the title to remove the word "bias" and added a new paragraph about this to the Discussion section.

The linear models on lines 526 and 542 should include an error term.

An error term has been added.

The description of the datasets appears twice in "Data Sources and data retrieval" and "Availability of data and materials."

The duplication has been removed.

Reviewer #3: I think the authors have made important discoveries, however these discoveries can be interpreted better.
Here are suggestions to strengthen the manuscript:
1. The paper has been comparing TPM with TMM, which I do not think it is necessary. TPM is calculated by double normalization of gene length and library size. Essentially, it is a proportion estimate but not a quantity estimate. The metric can be used for within-sample comparison or clustering analysis. It is not a legitimate metric for cross-sample quantitative analysis. If a study has used TPM for eQTL analysis, that should consider as a common mistake. It is not appropriate to compare and benchmark with wrong metrics in a research paper, given that you already know its drawbacks. I suggest to include values normalized by median of ratios for comparison instead, as TMM takes into account length but median of ratios doesn't.

We have moved the TMM-based analysis to the main text in order to emphasize those, and moved TPM-based results to the supplementary material. Median of ratios is similar to TMM except for not taking into account gene length, which we feel is an important factor to include in normalization, so we have opted to focus solely on TMM.

2. It is not surprising that the transcriptome diversity is associated with PEER factors from bulk RNA-seq. In bulk RNA-seq, the transcripts are amplified during sequencing process, where they compete for chance for amplification. GC content, gene length and abundance are all factors that can determine how many of them get the slots during this stochastic process. But the primary contributor will be their relative abundances. This is also the reason that read count of a gene depends on all other genes. The degrees of freedom constrained by limited sequencing depth will naturally lead to our observed association between the transcriptome diversity and read counts. I suspect such association will not be as strong in data with Unique Molecule Identifiers (UMIs), such as some of single cell RNA-seq data, where the amplification bias has been mitigated by these identifiers.

Thanks for the comment. We added the following text in the manuscript to discuss about datasets with UMI:

“It is worth noting that all RNA data analyzed in this study originates from bulk sequencing where reads do not include a unique molecular identifier (UMI). The nature of this data makes it prone to be influenced by experimental artifacts during library preparation, for example PCR amplification leading to inaccurate transcript estimates. It is possible that transcriptome diversity is influenced by such artifacts. Applying the analyses presented in this study to single-cell RNA-seq data with UMIs would shed light on the influence of library prep artifacts on transcriptome diversity.”

3. The transcriptome diversity is manifestation of different regulation and functions of cell types. Besides technical factors, it should be confounded with biological factors such as cell types, developmental stages, transcriptional phase, genders, to name a few. That is to say, we cannot correct for the transcriptome diversity, otherwise we remove important biological variation. Although it can explain large portion of PEER factors, the metric itself does not dissect whether that portion is biological or technical. I suggest to regress PEER factors to biological factors and technical factors (sequencing depth in particular) to evaluate their contributions.

We have regressed out some biological factors (sex) and technical factors (platform, PCR) from PEER factors in Fig 5b, and we now added ‘sequencing depth’ as a technical factor in the analysis in Fig 5b. The result shows that out of all these factors, transcriptome diversity accounts for the most PEER covariate variance.
