# Supplementary material for: Transcriptome diversity is a systematic source of variation in RNA-sequencing data
Source: PLoS Comput Biol. 2022 Mar 24;18(3):e1009939. doi: 10.1371/journal.pcbi.1009939 (PMC8982896; doi:10.1371/journal.pcbi.1009939)
Supplement: Attachment — Submitted filename: PLOS review_0201.docx [file pcbi.1009939.s009.docx]

Reviewer #1: Line 10, “it is still challenging to detect and remove artifactual signals”: this statement may be inaccurate given the existence of methods such as PEER.

We have modified this statement as: “it is still challenging to interpret and remove artifactual signals”.

Line 30, “This is roughly similar to a doctor that treats only symptoms of a disease, since she does not know the underlying cause. Understanding the cause is not enough to cure the disease, but is a critical first step”: This analogy should be removed, in my opinion.

We have removed this sentence.

Line 91, “PEER factors a priori are not associated with any known biological or technical source”: they are associated, just not super strongly.

We have modified this statement as: “PEER factors a priori are not strongly associated with any known biological or technical source”.

Line 115, “transcriptome diversity as measured by Shannon entropy [14] (Fig. 1; see Methods) across RNA-seq samples strongly correlates with the expression of many genes (measured by TMM)”: your Shannon entropy is calculated within each sample, across genes (not across samples).

We have modified this statement as: “transcriptome diversity as measured by Shannon entropy [14] (Fig. 1; see Methods) strongly correlates with the expression of many genes (measured by TMM)”.

Line 128, “We observed that most TMM-based expression values were correlated with transcriptome diversity (68.9% of genes at Benjamini-Hochberg False Discovery Rate (BHFDR) <0.05; see Methods). Interestingly, most (64%) of the significant correlations are positive”: it would be helpful to somehow indicate the significant correlations in the plot.

We have modified Fig 2 by adding a plot as Fig 2b to indicate the significant correlation in the plot.

Line 170, “To our knowledge this is the first example of a known source of variability explaining PEER covariates to such an extent”: in GTEx data, how much variance do the known covariates explain? That info could be included here for comparison.

We have shown other covariates including sex, sequencing depth, sequencing platform, PCR amplification and the first 5 PCs from the genotype matrix, explain much lower variance compared to transcriptome diversity across tissues in GTEx data in Fig 5b and supplementary material (Additional file 3: Table S2). We moved this sentence to Line 243 to better aid the interpretation of this statement.

Response to previous comments, “We now define p_i in terms of TPM in the main text. We do not think it would be ideal to define p_i using TMM since this across-sample normalization would make the transcriptome diversity values depend on what other samples are being included and therefore more difficult to interpret”: this does not make sense. If the authors believe entropy shouldn’t be calculated based on TMM values, then how do the authors justify calculating entropy based on TMM values throughout the paper?

We apologize for the lack of clarity; entropy is based on TPM rather than TMM. We followed the definition of Shannon Entropy to calculate transcriptome diversity and p_i is strictly defined as the probability of observing a read from a gene in a sample. We have shown in the supplementary material (Additional File 4: Note S1) that this probability is properly derived from TPM. We only use TMM in downstream analysis of entropy values.

Response to previous comments, “We did try reordering this figure by sample size (shown below), but we didn’t see any clear pattern”: the tissue types should still be ordered by sample size to demonstrate there is no pattern, in my opinion.

We now added a sentence to the caption of Fig. 4a, and a figure to supplementary material, to state that there is no pattern if ordered by sample size. “Tissues are ordered by absolute Spearman correlation coefficient, and there is no pattern observed when ordered by sample size (Additional File 1: Fig. S2).”
